# *BMC Psychiatry* reviewer acknowledgement 2014

**DOI:** 10.1186/s12888-015-0396-y

**Published:** 2015-03-21

**Authors:** Catherine M Rice, Diana M Marshall

**Affiliations:** BioMed Central, Floor 6, 236 Gray’s Inn Road, London, WC1X 8HB UK

## Abstract

The editors of *BMC Psychiatry* would like to thank all our reviewers who have contributed to the journal in Volume 14 (2014).

**Eivind Aakhus**

Norway

**Giovanni Abbate-Daga**

Italy

**Chadi Abdallah**

USA

**Riadh Abed**

UK

**Marielle Abrahamse**

Netherlands

**Ashley Acheson**

USA

**Marcel Aebi**

Switzerland

**Brian Ahmedani**

USA

**Christopher Ahnallen**

USA

**Kirsi Ahola**

Finland

**Shahin Akhondzadeh**

Iran

**Chelsea Ale**

USA

**Shiri Altman**

Israel

**Paula Alves**

Portugal

**Benedikt Lorenz Amann**

Spain

**Bart Ament**

Netherlands

**Federico Amianto**

Italy

**Clare Anderson**

Australia

**Alessandro Angrilli**

Italy

**Jules Angst**

Switzerland

**Kurt Angstman**

USA

**Trevor Archer**

Sweden

**Iris Arends**

Netherlands

**Todd Arnedt**

USA

**Hakan Atalay**

Turkey

**Anna Rita Atti**

Italy

**Dana Atzil Slonim**

Israel

**Carlos Ayan**

Spain

**Sofie Bäärnhielm**

Sweden

**Enrique Baca-Garcia**

Spain

**Dusan Backovic**

Serbia

**Imke Baetens**

Belgium

**Amber Bahorik**

USA

**Gianluca Baio**

UK

**Maarten Bak**

Netherlands

**Amanda Baker**

Australia

**Eleanor Bantry White**

Ireland

**Yoram Barak**

Israel

**Mariapaola Barbato**

Canada

**Izabela Barbosa**

Brazil

**Nicola Barclay**

UK

**Rachel Barnes**

USA

**Stefano Baroni**

Italy

**Giovanni Battista Bartolucci**

Italy

**Judith Bass**

USA

**Deborah Bassett**

USA

**Daniel Bateman**

USA

**Stephanie Bauer**

Germany

**Thomas Beblo**

Germany

**Margherita Bechi**

Italy

**Arthur Becker-Weidman**

USA

**Hassan Bella**

Saudi Arabia

**Martino Belvederi Murri**

Italy

**Raoul Belzeaux**

France

**Susan Mary Benbow**

UK

**Maneeton Benchalak**

Thailand

**Kristina Bennert**

UK

**Dror Ben-Zeev**

USA

**Yemane Berhane**

Ethiopia

**Kalpana Bhaskaran**

Singapore

**Kamaldeep Bhui**

UK

**Andargachew Kassa Biratu**

Ethiopia

**Victoria Bird**

UK

**Carl Birmingham**

Canada

**Istvan Bitter**

Hungary

**Simon Blackwell**

UK

**Sergio Blay**

Brazil

**Olof Blix**

Sweden

**D Blumberger**

Canada

**Sune Bo**

Denmark

**Jed Boardman**

UK

**Petr Bob**

Czech Republic

**Julio Bobes**

Spain

**Iwona Bojar**

Poland

**Ilaria Bonoldi**

UK

**Rhonda Booth**

UK

**Rami Bou Khalil**

France

**Aurore Boulard**

Belgium

**Jennifer Boyd**

USA

**Laurent Boyer**

France

**Lindy-Lou Boyette**

Netherlands

**Abby Braden**

USA

**Joanne Brady**

USA

**Nicholas Breitborde**

USA

**Benjamin Brent**

USA

**David Brent**

USA

**Joshua Breslau**

USA

**Jeffrey Bridge**

USA

**William Brinkman**

USA

**Teresita Briones**

USA

**Markus Britschgi**

Switzerland

**June Brown**

UK

**Margaret Brown**

Australia

**Martin Bruene**

Germany

**Scott Brunero**

Australia

**Andre Brunoni**

Brazil

**Fabrizio Bruschi**

Italy

**Kelly Buck**

USA

**Kyle Burghardt**

USA

**Julius Burkauskas**

Lithuania

**Lisa Burke**

Australia

**Christopher Bushe**

UK

**Andrew Bushmakin**

USA

**Casimiro Cabrera Abreu**

Canada

**Raffaele Cacciaglia**

Spain

**John Cacioppo**

USA

**Kristin Cadenhead**

USA

**Joseph Calabrese**

USA

**Alison Calear**

Australia

**Michael Caligiuri**

USA

**John Cape**

UK

**Alejandra Caqueo-Urizar**

Chile

**R. Nicholas Carleton**

Canada

**Hannah Carliner**

USA

**Vaughan Carr**

Australia

**Giovanni Castellini**

Italy

**Carl Castro**

USA

**Ferrán Catalá-López**

Spain

**Pascal Cathebras**

France

**Hilal Ceit**

Netherlands

**Eduardo Chachamovich**

Canada

**Subhajit Chakravorty**

USA

**Lai Fong Chan**

Malaysia

**Raymond Chan**

China

**Kristine Chapleau**

USA

**Robert Chaplin**

UK

**Gregory Chasson**

USA

**Imran Chaudhry**

UK

**Huafu Chen**

China

**Chih-Ying Chen**

USA

**Manoranjenni Chetty**

UK

**Dixon Chibanda**

Zimbabwe

**Howard Chilcoat**

USA

**Weng Yee Chin**

Hong Kong

**Dan Chisholm**

Switzerland

**Pratap Chokka**

Canada

**Yuan-Hwa Chou**

Taiwan

**David Christmas**

UK

**Quetzal Class**

USA

**Claude Robert Cloninger**

USA

**Laurie Clune**

Canada

**Vanessa Cobham**

Australia

**Alex Cohen**

UK

**Moran Cohn**

Netherlands

**Hannie Comijs**

Netherlands

**David Conn**

Canada

**Amy Conrad**

USA

**Andrew Cook**

USA

**Maurizio Coppola**

Italy

**Rhiannon Corcoran**

UK

**Angel Correa**

Spain

**Deborah Corring**

Canada

**Samuele Cortese**

USA

**William Coryell**

USA

**Philippe Courtet**

France

**Kay Lorraine Cox**

Australia

**Paul Crits-Christoph**

USA

**Anselm Crombach**

Germany

**Gary Cuddeback**

USA

**Alexis Cullen**

UK

**Kathryn Cullen**

USA

**Larry Culpepper**

USA

**Caroline Dalton**

UK

**Gerhard Dammann**

Switzerland

**Louise Danielsson**

Sweden

**Alison Darcy**

USA

**Carl D'Arcy**

Canada

**Renaud David**

France

**Larry Davidson**

USA

**Darlene Davis**

USA

**Derek De Beurs**

Netherlands

**Nicola De Carlo**

Italy

**Giovanni De Girolamo**

Italy

**Peter De Jonge**

Netherlands

**Flora De La Barra**

Chile

**Jose De Leon**

USA

**Jesus De Pedro-Cuesta**

Spain

**Varuni De Silva**

Sri Lanka

**Nicholas Deakin**

UK

**Catherine Deeprose**

UK

**Ryan Delapp**

USA

**Philippe Delespaul**

Netherlands

**Chao Deng**

Australia

**Joseph Deng**

USA

**Niklaus Denier**

Switzerland

**Birgit Derntl**

Germany

**Alain Dervaux**

France

**David Diamond**

USA

**Albert Diefenbacher**

Germany

**Danai Dima**

UK

**Steven Dobscha**

USA

**Tara Donker**

Australia

**Sarah Doucette**

Canada

**Darin Dougherty**

USA

**Irena Draskovic**

Netherlands

**Boris Drozdek**

Netherlands

**Fei Du**

USA

**Ke-Lin Du**

China

**Arnaud Duhoux**

Canada

**Sophie Duranceau**

Canada

**Nicola Dusi**

Italy

**John Dziak**

USA

**Bjorn Ebdrup**

Denmark

**Lara Ebenfeld**

Germany

**David Daniel Ebert**

Germany

**Chad Ebesutani**

Korea, South

**Takashi Ebisawa**

Japan

**Thomas Ehring**

Germany

**Solvig Ekblad**

Sweden

**Thomas Elbert**

Germany

**Jennifer Elliott**

USA

**Graham Emslie**

USA

**Jerome Endrass**

Switzerland

**Harald Engler**

Germany

**Linda Ercoli**

USA

**Pascale Esch**

Luxembourg

**Ozlem Eylem**

Netherlands

**Harris Eyre**

Australia

**Sonya Faber**

Germany

**Xiang Fan**

USA

**Xiaoduo Fan**

USA

**Aida Farreny**

Spain

**Ken Farrington**

UK

**Secondo Fassino**

Italy

**Johannes Fellinger**

Austria

**Liang Feng**

Singapore

**Christopher Ferguson**

USA

**Jilles Fermont**

UK

**Ana Fernandez**

Spain

**Maria Isabel Fernández San Martín**

Spain

**Fernando Fernandez-Aranda**

Spain

**Mark Ferro**

Canada

**Mathew Fetzner**

Canada

**Jess Fiedorowicz**

USA

**Andrea Fiorillo**

Italy

**Sarah Fischer**

USA

**Ellen Fitzsimmons-Craft**

USA

**Amos Fleischmann**

Israel

**Marie-Josee Fleury**

Canada

**Leon Flicker**

Australia

**Tim Fong**

USA

**Eduardo Fonseca Pedrero**

Spain

**David Forbes**

Australia

**Thomas Forkmann**

Germany

**Tomislav Franic**

Croatia

**Kenneth Freedland**

USA

**Richard Frye**

USA

**Sanae Fukuda**

Japan

**Sadaaki Fukui**

USA

**Daniel Fung**

Singapore

**Adel Gabriel**

Canada

**Patrick Gajewski**

Germany

**Gilad Gal**

Israel

**Silvia Gallagher**

Ireland

**Juan Gallego**

USA

**Orsola Gambini**

Italy

**Matthew Gambino**

USA

**David Garcia**

Switzerland

**Ricardo García-Mayor**

Spain

**Genevieve Gariepy**

Canada

**Andrew Garratt**

Norway

**Faye Gary**

USA

**Fiona Gaughran**

UK

**Caterina Gawrilow**

Germany

**Measho Gebregziabher**

Ethiopia

**James Gedra**

USA

**Bizu Gelaye**

USA

**Dejan Georgiev**

UK

**Adam Gerace**

Australia

**Adam Geraghty**

UK

**Lilian Ghandour**

Lebanon

**Lucio Ghio**

Italy

**Domenico Giacco**

UK

**Katrin Giel**

Germany

**Itzhak Gilat**

Israel

**Stephen Gilman**

USA

**Damiano Girardi**

Italy

**Ragy Girgis**

USA

**Lydia Gisle**

Belgium

**Melissa Gladstone**

UK

**Heide Glaesmer**

Germany

**Joseph Glass**

USA

**Robert Göder**

Germany

**Betty Goguikian Ratcliff**

Switzerland

**Carlos Gois**

Portugal

**Lutz Goldbeck**

Germany

**David Goldberg**

UK

**Hadass Goldblatt**

Israel

**Andrea Goldschmidt**

USA

**Jesus Gomar**

USA

**Marcus Gomes**

Brazil

**Rafael Gonzalez**

UK

**Robert Gonzalez**

USA

**Alexandre Gonzalez-Rodriguez**

Spain

**Peter Goossens**

Netherlands

**Jessica Goren**

USA

**Carla Maria Gramaglia**

Italy

**Iria Grande**

Spain

**Michael Grandner**

USA

**Kelly Green**

USA

**Tracy Greer**

USA

**Sebastien Grenier**

Canada

**James Griffith**

USA

**Tamasine Grimes**

Ireland

**Sandeep Grover**

India

**Anne Grundy**

Canada

**Martin Grunwald**

Germany

**Heinz Grunze**

UK

**Maria Rosaria Gualano**

Italy

**Cristiana Guetti**

Italy

**Mai-Britt Guldin**

Denmark

**Emilio Gutierrez**

Spain

**Yari Gvion**

Israel

**Noami Hadas- Lidor**

Israel

**Mark Haddad**

UK

**Hyeouk Chris Hahm**

USA

**Helinä Hakko**

Finland

**Mark Hamer**

UK

**David Hanauer**

USA

**Tonelle Handley**

Australia

**Lars Hansson**

Sweden

**Sheila Hardy**

UK

**Michelle Harley**

Ireland

**Ashley Harrison**

USA

**Christopher Harte**

USA

**Sigan Hartley**

USA

**Daniel Hartung**

USA

**Carol Harvey**

Australia

**Kenji Hashimoto**

Japan

**Ilanit Hasson-Ohayon**

Israel

**Tobias Hecker**

Germany

**Craig Anne Heflinger**

USA

**Markus Heinimaa**

Finland

**Martin Hellmich**

Germany

**Claire Henderson**

UK

**Urs Hepp**

Switzerland

**Andres Herane Vives**

UK

**Philipp Herzberg**

Germany

**Klaus Hesse**

Germany

**Morten Hesse**

Denmark

**Terrence Hill**

USA

**Hubertus Himmerich**

Germany

**Andreas Hinz**

Germany

**Erin Hoare**

Australia

**Robert Hodapp**

USA

**Nicolas Hoertel**

France

**Michael Höfler**

Germany

**Tobias Hofmann**

Germany

**Marjan Holloway**

USA

**Mikael Holma**

Finland

**Sarah Honaker**

USA

**Lisa Horowitz**

USA

**Samantha Horswill**

Canada

**Chiung-Yu Huang**

Taiwan

**Teresa Hudson**

USA

**Libby Hughes**

Australia

**Annemiek Huisman**

Netherlands

**Benjamin Hummelen**

Norway

**Rene Hurlemann**

Germany

**Tuula Hurtig**

Finland

**Gerard Hutchinson**

Trinidad and Tobago

**Mari Hysing**

Norway

**Felice Iasevoli**

Italy

**Benjamin Iffland**

Germany

**Yoshito Igarashi**

Japan

**Masumi Iida**

USA

**Masatoshi Inagaki**

Japan

**Pasquale Innominato**

France

**Makoto Ishitobi**

Japan

**Mariko Iwayama**

Japan

**Nadja Jacob**

Switzerland

**Sabrina Anne Jacob**

Malaysia

**Frank Jacobi**

Germany

**Susanne Jaeger**

Germany

**Anthony James**

UK

**Josee L Jarry**

Canada

**Rachel Jenkins**

UK

**Tawanchai Jirapramukpitak**

Thailand

**Stefan Johansson**

Norway

**Bjorn Johnson**

Sweden

**Mandy Johnstone**

UK

**Sheila Jones**

Denmark

**Ruud Jongedijk**

Netherlands

**Andreas Joos**

Germany

**Rikke Jørgensen**

Denmark

**Georg Juckel**

Germany

**Minyoung Jung**

Korea, South

**Mario Juruena**

Brazil

**Colette Kabrita**

Lebanon

**Michael Kaess**

Germany

**Kirsti Kähärä**

Finland

**Ondrej Kalina**

Slovakia

**Bengt Källén**

Sweden

**Eva Kaltenthaler**

UK

**Inge Kamp-Becker**

Germany

**Olli Kampman**

Finland

**Richard Antony Alexander Kanaan**

UK

**Yukiko Kano**

Japan

**Joshua Kantrowitz**

USA

**Manav Kapoor**

USA

**Nestor Damian Kapusta**

Austria

**Nilamadhab Kar**

UK

**Susanne Karch**

Bosnia and Herzegovina

**Andrea Kass**

USA

**Takahiro Kato**

Japan

**Marcia Kauer-Sant'Anna**

Brazil

**Brooke Kauffman**

USA

**Anne Kavanagh**

Australia

**Yoshitaka Kawashima**

Japan

**Robert Keeley**

USA

**Matthew Keller**

USA

**Friederike Kendel**

Germany

**Harry Kennedy**

Ireland

**Martin Kennedy**

New Zealand

**Michelle Kermode**

Australia

**Sarah Kertz**

USA

**Alice Keski-Valkama**

Finland

**Ronald Kessler**

USA

**Hari-Mandir Khalsa**

USA

**Judi Kidger**

UK

**Jae-Min Kim**

Korea, South

**Yong Sik Kim**

Korea, South

**David Kingdon**

UK

**John David Kinzie**

USA

**Julia Kirkham**

Canada

**Anne Kleinsasser**

USA

**Elizabeth Klerman**

USA

**Sören Kliem**

Germany

**Stefan Kloiber**

Germany

**Christine Knaevelsrud**

Germany

**Jeroen Knipscheer**

Netherlands

**Sarah Knowles**

UK

**Kathrin Koch**

Germany

**Manami Kodaka**

Japan

**Stephan Köhler**

Germany

**Michael Kohn**

Australia

**Tsuyoshi Kondo**

Japan

**Barna Konkoly Thege**

Canada

**Dimitrios Kontis**

Greece

**Hans Koppeschaar**

Netherlands

**Jyrki Korkeila**

Finland

**Hirotaka Kosaka**

Japan

**Ernst Koster**

Belgium

**Diana Koszycki**

Canada

**Leda Kovatsi**

Greece

**Kate Krajci**

USA

**Ueli Kramer**

Switzerland

**Levente Kriston**

Germany

**Kurt Kroenke**

USA

**Jesper Krogh**

Denmark

**Ziad Kronfol**

Qatar

**Hans Kroon**

Netherlands

**Tillmann Kruger**

Germany

**Robert Kumsta**

Afghanistan

**Matthew Kurtz**

USA

**Philipp Kuwert**

Germany

**Jeoung A Kwon**

Korea, South

**Cornbelis Laban**

Netherlands

**Jean Lachaine**

Canada

**Daniel Lai**

Canada

**Brian Lakey**

USA

**Marie-Eve Lamontagne**

Canada

**Carol Landis**

USA

**Nils Inge Landrfø**

Norway

**Pierre Lau**

Belgium

**Chi-Kin Law**

Hong Kong

**Robyne Le Brocque**

Australia

**Monique Lebourgeois**

USA

**Daniel Lebouthillier**

Canada

**David Ledgerwood**

USA

**William Lee**

UK

**Robert Leeman**

USA

**Seblewengel Lemma**

Ethiopia

**Bethany Leonhardt**

USA

**Karolina Leopold**

Germany

**Stefan Leucht**

Germany

**Catharine J. Lewis**

Germany

**Jun Li**

China

**Kaigang Li**

USA

**Pesach Lichtenberg**

Israel

**Belinda Liddell**

Australia

**Shih-Ku Lin**

Taiwan

**Jean-Pierre Lindenmayer**

USA

**Jamey Lister**

USA

**Xianhua Liu**

China

**Zhongchun Liu**

China

**James Livingston**

Canada

**Brynmor Lloyd-Evans**

UK

**Alexandre Loch**

Brazil

**Steven Lockley**

USA

**Paul Lombroso**

USA

**Barbara Lopes Cardozo**

USA

**Jorge Lopez-Castroman**

Spain

**Lee-Fay Low**

Australia

**Evan Lutkenhoff**

USA

**Sean Lynch**

UK

**John Lyne**

Ireland

**Corey Mackenzie**

Canada

**Helen Mactier**

UK

**Franziska Maier**

Germany

**Thomas Maier**

Switzerland

**Pallab Majumder**

UK

**Marelign Malaju**

Ethiopia

**Sumaya Mall**

USA

**Jerome Maller**

Australia

**Stefania Mannarini**

Italy

**Donatella Marazziti**

Italy

**Angelo Giovanni Icro Maremmani**

Italy

**Dominik Marin**

Germany

**Michel Maron**

France

**Colin Martin**

UK

**Leticia Martínez González**

Spain

**Jose Martinez-Raga**

Spain

**Francesca Martino**

Italy

**Matteo Martino**

Italy

**Enrica Marzola**

Italy

**Susan Marzolini**

Canada

**Holly Mash**

USA

**Julie Maslowsky**

USA

**Davide Massidda**

Italy

**Aleksandra Matanov**

UK

**Faith Matcham**

UK

**Catherine Mathews**

South Africa

**Yuki Matsumoto**

Japan

**Fermin Mayoral**

Spain

**Patrick Mccabe**

USA

**Rosemarie Mccabe**

UK

**Terence Mccann**

Australia

**Kevin Mccarthy**

USA

**Troy Mcewan**

Australia

**Gary Mclean**

UK

**Hamish Mcleod**

UK

**Caitlin Mcomish**

Australia

**Melissa Mcpheeters**

USA

**Nick Meader**

UK

**Denis Meadows**

Australia

**Ybe Meesters**

Netherlands

**Alison Menatti**

USA

**Itiana Castro Menezes**

Brazil

**Andreas Menke**

Germany

**Fiona Mensah**

UK

**Lisa Merlo**

USA

**Thomas Messer**

Germany

**Ricarda Mewes**

Germany

**Patrick Michaels**

USA

**Makoto Michikawa**

Japan

**Daniela Mier**

Germany

**Jouko Miettunen**

Finland

**Rafael T Mikolajczyk**

Germany

**Brian Miller**

USA

**Alessandra Minelli**

Italy

**Silvia Minozzi**

Italy

**Regina Miranda**

USA

**Mansha Mirza**

USA

**Mitsuhiro Miyashita**

Japan

**Toshiki Mizuno**

Japan

**Shakeh Moamrtin**

Australia

**Sara Modig**

Sweden

**Roy Moncayo**

Austria

**Akira Monji**

Japan

**Christiane Montag**

Germany

**Ali Montazeri**

Iran

**Galia Moran**

Israel

**Carlos Renato Moreira Maia**

Brazil

**Kevin Morgan**

UK

**Elaine Morrato**

USA

**Richard Morriss**

UK

**Anett Mueller-Alcazar**

Germany

**Gerry Mugford**

Canada

**Amin Muhammad Gadit**

Canada

**Matthias J Müller**

Germany

**Astrid Müller**

Germany

**Gioia Mura**

Italy

**Stuart Murray**

Australia

**Jochen Mutschler**

Switzerland

**Maria Muzik**

USA

**Inez Myin-Germeys**

Netherlands

**Makalani Myrtveit**

Norway

**Michael Nadorff**

USA

**Farooq Naeem**

Pakistan

**Akiko Nakagawa**

Japan

**Takashi Nakamae**

Japan

**Hideyuki Nakane**

Japan

**Ora Nakash**

Israel

**Kristin Naragon-Gainey**

USA

**Davide Nardo**

Italy

**Carryl Navalta**

USA

**Manuela Neuman**

Canada

**Kelly Newell**

Australia

**James Newham**

UK

**Christian Hans Nickel**

Switzerland

**Angela Nickerson**

USA

**Inga Niedtfeld**

Germany

**Dana Jh Niehaus**

South Africa

**Katsuji Nishimura**

Japan

**Maria Nobile**

Italy

**Egil Nygaard**

Norway

**Shwe Zin Nyunt**

Singapore

**Michael Obermeier**

Germany

**Louise O'Brien**

USA

**Daniel O'Connor**

Australia

**Bridianne O'Dea**

Australia

**Michael Gerhard Odenwald**

Germany

**Brian O’Donoghue**

Australia

**Jeneva Ohan**

Australia

**Takashi Okada**

Japan

**Yasuyuki Okumura**

Japan

**Francesco Oliva**

Italy

**Javier Oltra-Cucarella**

Spain

**Joyce O'Mahony**

Canada

**Sepideh Omidvari**

Iran

**Juliana Onwumere**

UK

**Claire O'Reilly**

Australia

**Vasiliki Orgeta**

UK

**Killian O'Rourke**

Ireland

**Maciej Owecki**

Poland

**Shane Owens**

USA

**Yuji Ozeki**

Japan

**Seline Ozer**

UK

**Chi-Un Pae**

Korea, South

**Andrew Page**

Australia

**Daniel Pagnin**

Brazil

**Utpal Pajvani**

USA

**Ståle Pallesen**

Norway

**Maria Panagioti**

UK

**Ananda Pandurangi**

USA

**Igor Pantic**

Serbia

**A-La Park**

UK

**Sohee Park**

USA

**Barbara Parry**

USA

**Timo Partonen**

Finland

**Augusto Pasini**

Italy

**Sue Patterson**

Australia

**Lenin Pavon**

Mexico

**Nalin Payakachat**

USA

**Martha Payne**

USA

**Carsten Bøcker Pedersen**

Denmark

**Kirsi Peltonen**

France

**Cinzia Perlini**

Italy

**Jane Persons**

USA

**Inge Petersen**

South Africa

**Anett Pfeiffer**

Uganda

**Andrea Pfennig**

Germany

**Ruth Pidsley**

Australia

**Anilkumar Pillai**

USA

**Martin Pinquart**

Germany

**Melissa Pinto**

USA

**Yehuda Pollak**

Israel

**Nunzio Pomara**

USA

**Maurizio Pompili**

Italy

**Alexander Ponizovsky**

Israel

**Piero Porcelli**

Italy

**Maj-Britt Posserud**

Norway

**Robert Powers**

USA

**Sarah Pratt**

USA

**Peter Pregelj**

Slovenia

**Antonio Preti**

Italy

**Stefan Priebe**

UK

**Petroula Proitsi**

UK

**David Pulsford**

UK

**Stephen Puntis**

UK

**Marianna Purgato**

Italy

**Bernd Puschner**

Germany

**Eli Puterman**

USA

**Terry Quinn**

UK

**Daniel Racey**

UK

**Mikael Rahmqvist**

Sweden

**Mahesh Rajasuriya**

Sri Lanka

**Anto P. Rajkumar**

Denmark

**Padmavati Ramachandran**

India

**Hugh Ramsay**

Ireland

**Beverley Raphael**

Australia

**Carla Rash**

USA

**Silvia Ravera**

Netherlands

**Ursula Read**

UK

**Nicola Reavley**

Australia

**Elliott Rees**

UK

**Susan Rees**

Australia

**Gayle Reiber**

USA

**Kathryn Reid**

USA

**Lucia Reisch**

Denmark

**Javier Rejas**

Spain

**Solomon Renati**

India

**Leslie Rescorla**

USA

**Petra Retz-Junginger**

Germany

**Valdo Ricca**

Italy

**Derek Richards**

Ireland

**Debra Rickwood**

Australia

**Philipp S. Ritter**

Germany

**Patricio Riva-Posse**

USA

**Brandy Roane**

USA

**Laurence Inès Martine Robel**

France

**Pasquale Roberge**

Canada

**David Roberts**

USA

**Seren Roberts**

UK

**Jo Robinson**

Australia

**Dan Robotham**

UK

**James Roerig**

USA

**Paul Rohde**

USA

**Suely Roizenblatt**

Brazil

**Maria Graciela Rojas Castillo**

Chile

**Martha Romero**

Mexico

**Roberto Rona**

UK

**Kathlyn Ronaldson**

Australia

**Rita Roncone**

Italy

**Jennifer Rose**

USA

**Roderick Rose**

USA

**Irwin Rosenfarb**

USA

**Daniel Rossignol**

USA

**Francesco Rotella**

Italy

**Cecile Rousseau**

Canada

**Guy Rousseau**

Canada

**Michael Roy**

USA

**Abraham Rudnick**

Canada

**Montserrat Rue**

Spain

**Reiner Rugulies**

Denmark

**M Angeles Ruiperez**

Spain

**Miguel Ruiz-Veguilla**

Spain

**Dan Rujescu**

Germany

**Zlatka Russinova**

USA

**Jennifer Rusted**

UK

**Torleif Ruud**

Norway

**Joanne Ryan**

Australia

**Janusz Rybakowski**

Poland

**Andrew Ryder**

Canada

**Ad Sad**

Bahrain

**Hans Inge Sævareid**

Norway

**Yoichi Sakakihara**

Japan

**Joao Salgado**

Brazil

**Raimo K. R. Salokangas**

Finland

**Harshal Salve**

India

**Virginio Salvi**

Italy

**Zainab Samaan**

Canada

**Stephen Sammut**

USA

**Marsal Sanches**

USA

**Marco Sarchiapone**

Italy

**Jerome Sarris**

Australia

**Tsuyoshi Sasaki**

Japan

**Anna Sasdelli**

Italy

**Cathrin Sauer**

Germany

**Broderick Sawyer**

USA

**Andrea Sboner**

USA

**Aart Schene**

Netherlands

**Jeffrey Scherrer**

USA

**Robert Schlack**

Germany

**Jann E Schlimme**

Germany

**Marc Schmid**

Switzerland

**Regien Schoemaker**

Netherlands

**Georg Schomerus**

Germany

**Daniel Schöttle**

Germany

**Beate M. Schulze**

Switzerland

**Jan Scott**

UK

**Bridie Scott-Parker**

Bahamas

**Dallas Seitz**

Canada

**Carmen Senra**

Spain

**Michael Sernyak**

USA

**Argentina Servin**

USA

**Emanuel Severus**

Germany

**Asim Shah**

USA

**Katherine Sharkey**

USA

**Ann Sharpley**

UK

**Taiwo Lateef Sheikh**

Nigeria

**Yukihiko Shirayama**

Japan

**Dvora Shmulewitz**

USA

**Michael Shuman**

USA

**Tianmei Si**

China

**Etienne Sibille**

USA

**Ingrid Sibitz**

Austria

**Cecilia Sighinolfi**

Italy

**Roberta Siliquini**

Italy

**Derrick Silove**

Australia

**Kang Sim**

Singapore

**Magenta Simmons**

Australia

**Claudia Simons**

Netherlands

**Jay Singh**

Norway

**Mark Sinyor**

Canada

**Bonnie Wm Siu**

Hong Kong

**Anna Smith**

UK

**Patrick Smith**

USA

**Rebecca Smith**

UK

**Shubulade Smith**

UK

**David G Smithard**

UK

**Marcel Smits**

Netherlands

**Moria Smoski**

USA

**Anne Söderlund**

Sweden

**Daya Somasundaram**

Sri Lanka

**Lin Sørensen**

Norway

**Julia Friederike Sowislo**

Switzerland

**Francesco Spallotta**

Germany

**Mona Srivastava**

India

**Paul St John-Smith**

UK

**Wouter Staal**

Netherlands

**David Stacey**

Australia

**Victoria Stanhope**

USA

**Jack Stevens**

USA

**Jon Stone**

UK

**Martin Strassnig**

USA

**Barbara Stringer**

Netherlands

**Brendon Stubbs**

UK

**Paula Suárez-Pinilla**

Spain

**Hajime Sueki**

Japan

**Koreaki Sugimoto**

Japan

**Sarah Sullivan**

UK

**Jaana Suvisaari**

Finland

**Takefumi Suzuki**

Japan

**Bengt Svensson**

Sweden

**Piotr Switaj**

Poland

**Andras Szekely**

Hungary

**Eskinder Tafesse**

USA

**Yueh-Ming Tai**

Taiwan

**Ricardo Taipa**

Portugal

**Mutsuko Takahashi**

Japan

**Taro Takeshima**

Japan

**Qingrong Tan**

China

**Ilaria Tarricone**

Italy

**Cumhur Tas**

Turkey

**Alvin Kuowei Tay**

Australia

**David Taylor**

UK

**Kate Tchanturia**

UK

**Michelle Teale Sapach**

Canada

**Ghazel Tellawi**

USA

**Henk Temmingh**

South Africa

**Martin Teufel**

Germany

**Michel Thibodeau**

Canada

**Henning Tiemeier**

Netherlands

**Nickolai Titov**

Australia

**Arun Tiwari**

Canada

**Carmine Tomasetti**

Italy

**Ulysses Torres**

Brazil

**Lurdes Tse**

Canada

**Wan-Ling Tseng**

USA

**Marco Tuccori**

Italy

**James Tung**

Canada

**Alyna Turner**

Australia

**Hiroyuki Uchida**

Japan

**Alp Ucok**

Turkey

**Rudolf Uher**

Canada

**Zsolt Unoka**

Hungary

**Dominique Valade**

France

**Tom Van Daele**

Belgium

**Geurt Van De Glind**

Netherlands

**Fons Van De Vijver**

Netherlands

**Marjan Van Den Akker**

Netherlands

**Rutger Jan Van Der Gaaag**

Netherlands

**Peggy Van Der Pol**

Netherlands

**Louk Van Der Post**

Netherlands

**Annemarie Van Elburg**

Netherlands

**Harm Van Marwijk**

Netherlands

**Bregje Van Spijker**

Australia

**Cornelia Van Uden-Kraan**

Netherlands

**Benjamin Van Voorhees**

USA

**Erik Vanderlip**

USA

**Gustavo Vazquez**

Argentina

**Per Vendsborg**

Denmark

**Peter Ventevogel**

Netherlands

**Lena Verdeli**

USA

**Marjolein Verhoeven**

Netherlands

**Jan Vevera**

Czech Republic

**Philippe Vincent**

Canada

**Benedetto Vitiello**

USA

**David Vogel**

USA

**Jen Vohs**

USA

**Katharina Voigt**

Germany

**Corrine Voils**

USA

**Eberhard Voit**

USA

**Umberto Volpe**

Italy

**Silke Von Esenwein**

USA

**Martin Voracek**

Austria

**Tracey Wade**

Australia

**Margda Waern**

Sweden

**Akio Wakabayashi**

Japan

**Fredrik A. Walby**

Norway

**Wei Wang**

China

**Yuan-Pang Wang**

Brazil

**Jia Wang**

China

**Qiang Wang**

China

**Norio Watanabe**

Japan

**Rafal Watrowski**

Germany

**Florian Weck**

Germany

**Peter M. Wehmeier**

Germany

**Aviv Weinstein**

Israel

**Abraham Weizman**

Israel

**David M Wellsted**

UK

**Benedict Weobong**

Ghana

**Perla Werner**

Israel

**Ruud Wetzels**

Netherlands

**Amanda Wheeler**

Australia

**Ross White**

UK

**Richard Whittington**

UK

**Johanna Wigman**

Netherlands

**Dirk Wildgruber**

Germany

**Sarah Wilker**

Germany

**Alishia Williams**

Australia

**Amanda Williams**

UK

**Monnica Williams**

USA

**Tony Winefield**

Australia

**Tracy Witte**

USA

**Katrin Woitecki**

Germany

**Owen Wolkowitz**

USA

**Miranda Wolpert**

UK

**Eugene Wong**

UK

**Jesse Wright**

USA

**Yin Wu**

UK

**Til Wykes**

UK

**Brian Wymbs**

USA

**Shifu Xiao**

China

**Kirsten Yaffe**

USA

**Amit Yamin**

Israel

**Chih Chieh Yang**

USA

**Yen Kuang Yang**

Taiwan

**Philip Yanos**

USA

**Shuqiao Yao**

China

**Norio Yasui-Furukori**

Japan

**Wai-Ying Wendy Yau**

USA

**Barbara Yawn**

USA

**Ksenija Yeeles**

UK

**Cheng-Fang Yen**

Taiwan

**Yuan Yong Gui**

China

**Ki-Bong Yoo**

Korea, South

**Nicole Yuan**

USA

**Kai Yuan**

China

**Xiaojun Yuan**

USA

**David Yusko**

USA

**Clement Zai**

Canada

**Yu-Feng Zang**

China

**Faika Zanjani**

USA

**Yuliya Zaytseva**

Germany

**Patrizia Zeppegno**

Italy

**Xiang Rong Zhang**

China

**Huiping Zhang**

USA

**Han Zhang**

China

**Bin Zhang**

China

**Jie Zhang**

USA

**Min Zhao**

China

**Jiang-Ning Zhou**

China

**Gang Zhu**

China

**Tim Ziermans**

Netherlands

**Stephan Zipfel**

Germany

**Phillip Zoladz**

USA

**Jon-Kar Zubieta**

USA

**Maria Victoria Zunzunegui**

Canada

